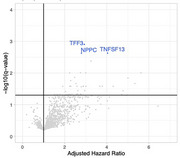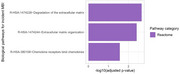# Plasma proteomic signatures of incident mild behavioral impairment – a longitudinal Singaporean memory clinic study

**DOI:** 10.1002/alz70856_106292

**Published:** 2026-01-08

**Authors:** Ming Ann Sim, Yingqi Liao, Cheuk Ni Kan, Hyung Won Choi, Arthur Mark Richards, Christopher Chen

**Affiliations:** ^1^ National University of Singapore, Singapore, Singapore, Singapore; ^2^ National University Hospital, Singapore, Singapore, Singapore; ^3^ Yong Loo Lin School of Medicine, National University of Singapore, Singapore, Singapore; ^4^ Memory, Ageing, and Cognition Centre (MACC), Department of Pharmacology, Yong Loo Lin School of Medicine, National University of Singapore, Singapore, Singapore; ^5^ Memory Aging and Cognition Center, National University Health System, Singapore, Singapore; ^6^ National University of Singapore, Singapore, Singapore; ^7^ Department of Medicine, Yong Loo Lin School of Medicine, National University of Singapore, Singapore, Singapore; ^8^ Christchurch Heart Institute, University of Otago, New Zealand, Christchurch, New Zealand; ^9^ Cardiovascular Research Institute, National University Health System, Singapore, Singapore, Singapore; ^10^ National University Health System, NUHS, Singapore, Singapore, Singapore; ^11^ Memory, Ageing and Cognition Centre, National University Health System, Singapore, Singapore

## Abstract

**Background:**

Mild behavioral impairment (MBI) is a diagnostic construct capturing neuropsychiatric symptom burden in pre‐dementia subjects, and has been increasingly recognized as an at‐risk entity for dementia [1,2]. However, prognostic biomarkers and mechanistic underpinnings of incident MBI remain unclear. We therefore utilized a Singaporean memory clinic cohort to evaluate the plasma proteomic signatures of incident MBI.

**Method:**

A Singaporean memory clinic cohort was followed‐up prospectively for 4 years. Dementia‐free subjects who did not have MBI at baseline were included for analysis. All subjects were assessed at baseline and annually using the Neuropsychiatric Inventory (NPI). The definition of incident MBI was made in alignment with the Society to Advance Alzheimer's Research and Treatment of the Alzheimer's Association (ISTAART) criteria, with incident MBI defined as the development of new neuropsychiatric symptoms (defined as an increment in NPI total score of ≥1) over any two consecutive years of follow‐up [3].

Plasma samples were obtained at baseline, and the Olink Explore platform was used to profile 1536 plasma proteins. The associations of plasma proteins with incident MBI were subsequently evaluated using multivariable cox regression models, accounting for multiple testing correction (*p*‐value < 0.05 and q‐value < 0.1).

**Result:**

Of 219 subjects included for analysis (mean age 71.1±7.6 years, 53% female, 66% hypertensive), 54 (24.7%) subjects developed incident MBI.

We identified 142 proteins (q‐value < 0.1, *p*‐value < 0.05) which significantly associated with the hazard of incident MBI after multivariate adjustment. The top 3 most associative proteins for incident MBI were TFF3, NPPC and TNFSF13 (Figure 1)

Overrepresentation analysis of all significant proteins revealed diverse biological pathways underpinning incident MBI. The top 3 most significantly over‐represented biological pathways included extracellular matrix disruption and immune dysregulation (Figure 2).

**Conclusion:**

We report plasma proteomic signatures and biological pathways of incident MBI. Future mechanistic studies are required to elucidate the utility of these markers as therapeutic targets for incident MBI and its cognitive sequelae.

**References**

1. Kan et al, J Clin Psychiatry (2022)

2 Ismail Z et al, J Alzheimers Dis (2021)

3 Ismail Z et al, J Alzheimers Dis (2017)